# Genetic and phenotypic study of methicillin-resistant *Staphylococcus aureus* among patients and health care workers in Mansoura University Hospital, Egypt

**Published:** 2017-04

**Authors:** Walaa Othman Elshabrawy, Maysaa Elsayed Zaki, Mohamed Farag Kamel

**Affiliations:** 1 Clinical Pathology Department, Mansoura Faculty of Medicine, Mansoura, Egypt; 2 Vascular Surgery Department, Mansoura Faculty of Medicine, Mansoura, Egypt

**Keywords:** MRSA, ChromID medium, Multiplex PCR

## Abstract

**Background and Objectives:**

*Staphylococcus aureus* is common pathogen that is associated with many hospital acquired infections. The virulence of *S. aureus* is identified with resistance to antibiotics especially to methicillin. Therefore the aims of the present study were to detect the carrier rates of methicillin-resistant *S. aureus* (MRSA) among health care Workers (HCWs) and patients and to compare use of specific chromogenic agar for MRSA culture with PCR for detection of MRSA genes.

**Materials and Methods:**

Samples obtained were subjected to full microbiological laboratory studies involving culture on specific chromogenic medium and antibiotics susceptibility testing for detection of MRSA and their resistance rates to other commonly used antibiotics. Furthermore multiplex PCR was carried out to detect *SCCmecA* genes.

**Results:**

*Staphylococcus aureus* was isolated from 70 (29.9%) of the studied subjects. MRSA isolates (n=28) had high resistance rates for the used antibiotics and the most common resistance was for ciprofloxacin and chloramphenicol (57.1% for each). MRSA was isolated mainly from health care workers (17.02%). The frequency of SCC*mecA* was 60.7% for type I, 25% for type III and 14.3% for type II. Chromogenic agar identified correctly MRSA isolates in 92.9%. PCR was positive in all isolates with resistance to cefoxitin disc.

**Conclusions:**

The present study highlights that MRSA carriage is common among health care workers in one Egyptian tertiary care hospital. The major genotype of MRSA is belonging to SCC*mecA* type I followed by type III and type II. ChromID medium is an accurrate culture method for detection of MRSA compared to molecular method.

## INTRODUCTION

*Staphylococcus aureus* is a common pathogen that is detected both in normal populations and isolated from several infections (1). There is an emergence of antimicrobial resistance strains especially to methicillin reported in multiple health care settings leading to higher morbidity and mortality associated especially with systemic methicillin-resistant *S. aureus* (MRSA) infections (2, 3).

Thus an importance arises to screen for the source of MRSA strains in hospitals especially among health care workers (HCWs) and to reduce its prevalence. The carrier of MRSA also has been shown to be associated with infections among HCWs in certain situations (4). Though these infections are mild studies have shown that it may turn to be chronic (5).

Thus surveillance of MRSA in health care settings is an important for good implementation of infection control policy. Laboratory detection of MRSA is an important method to confirm carrier states among patients and HCWs (6, 7) and the implementation of laboratory screening for MRSA is an important component of infection control program (8–11).

Genetic studies for MRSA identified structural genes responsible for methicillin resistance and it is known as *mecA* genes, also referred to as the staphylococcal chromosomal cassette gene SCC*mec*. There are three common types of SCC*mecA*, type I (34 kb), type II (52 kb), and type III (66 kb). More recently, a smaller fourth *mec* element, SCC*mec* type IV (20 to 24 kb), was independently identified among representatives of the pediatric clone and in two community-acquired MRSA strains (12, 13).

The use of selective media for rapid detection of MRSA is an important method with comparable reduced cost to more expensive method by molecular methods.

The chromogenic media use chromogenic substances incorporated into solid media that change in color due to presence of specific enzyme production by the target bacteria (14). The available media for MRSA use both chromogenic substances and antibiotics for differentiation of MRSA (15). ChromID detects the α-glucosidase enzyme of *S. aureus* by producing green colored colonies and inhibits non MRSA by presence of cefoxitin (4 mg/liter) (16, 17).

There are studies describing different chromogenic media for isolation of MRSA (16–21). However, these studies are usually laboratory based studies (16, 17) that’s to say not using clinical samples (18, 19) and not comparing the phenotypic detection of MRSA with molecular methods for detection of MRSA genotypes.

Therefore the aims of the present study were to detect the carrier rates of MRSA among HCWs and patients and to compare phenotypic methods by culture on specific chromogenic agar for MRSA with genotypic methods using polymerase chain reaction for detection of MRSA genes.

## MATERIALS AND METHODS

The study was carried out in Mansoura University hospital, Egypt between July 2015 till December 2015. Mansoura University hospital is a 1500 bed tertiary teaching hospital. The study included samples from health care workers and from patients admitted to different hospital departments. This cross sectional study was approved by Mansoura Faculty of Medicine ethical committee and approval written consents were obtained from the participants of the study. Sterile swabs moisten in sterile saline were used for sampling. From each subject two swabs were used one to obtain samples from both nostrils and the other swab for throat samples (combinations of sites increase the sensitivity for MRSA detection). Swabs were transported immediately to the laboratory.

### Culture methods.

Swabs were cultured on Mueller Hinton broth (Oxoid) with 6.5% NaCl for 24 hours at 37°C. Then subculture was performed on Columbia agar with 5% sheep blood (bioMérieux, France) and chromogenic medium MRSA-ID (bioMérieux, France) for 24 hours at 37°C. Colonies on both media were identified by Gram stainting, coagulase test and catalase reaction and color of colonies on ChromID agar were inspected for presence of green colonies. Purified colonies were obtained and stored at −20°C for further multiplex PCR for detection of SCC*mecA* genes.

### Cefoxitin disc screen test for detection of MRSA.

Susceptibility of *S. aureus* isolates to cefoxitin (30 μg) (BioRad, USA) was performed by the disc diffusion method on Mueller-Hinton agar plates (Oxoid) using a bacterial suspension equivalent to a 0.5 McFarland standard. Plates were incubated at 35°C for 24 hours. Results were interpreted according to CLSI (2013) guidelines (22). The interpretive criteria for cefoxitin were: *S. aureus*, sensitive ≥22 mm and resistant ≤21 mm.

*S. aureus* isolates resistant to cefoxitin disc were reported as MRSA. This is according to the recommendation of the CLSI that recommends the use of the cefoxitin that allows the growth of all MRSA strains (more potent inducer of *mecA* expression than other agents) and the inhibition of all methicillin-sensitive *S. aureus* (MSSA) strains and this test was validated in wide surveillance program (23).

### Antimicrobial susceptibility testing of MRSA.

The MRSA isolates were tested for antibiotic susceptibility by disc diffusion test using the following discs: chloramphenicol (30 μg), ciprofloxacin (5 μg), clindamycin (2 μg), fusidic acid (10 μg), gentamycin (10 μg), erythromycin (15 μg), imipenem (10 μg), rifampicin (5 μg), tetracycline (30 μg), trimethoprim/sulfamethoxazole (1.25 + 23.75 μg) and vancomycin (30 μg) (BioRad, USA). The rates of susceptibility and resistance of isolated MRSA strains to the used antibiotic discs were detected.

### Multiplex PCR for SCC*mecA* gene.

DNA was extracted from MRSA strains using DNA extraction kit (Qiagen, USA) according to the manufacturer’s instructions. The multiplex PCR was performed to detect three loci A, B and E of *mecA* gene for SCC-*mec* types I, II and III. The primers used are presented in Table 1.

**Table 1.** The sequence of primers used.

**Locus**	**Primer sequence**	**Amplicon size (bp)**	**SCC*mec* type**
A	CIF2 F2 [5′-TTCGAGTTGCTGATGAAGAAGG-3′]	495	I
	CIF2 R2 [5′-ATTTACCACAAGGACTACCAGC-3′]		
B	KDP F1 [5′-AATCATCTGCCATTGGTGATGC-3′]	284	II
	KDP R1 [5′-CGAATGAAGTGAAAGAAAGTGG-3′]		
E	RIF4 F3 [5′-GTGATTGTTCGAGATATGTGG-3′]	243	III
	RIF4 R9 [5′-CGCTTTATCTGTATCTATCGC-3′]		

The amplification reaction was carried out in 50 ul volume containing 5 ul of extracted DNA; 1x PCR buffer; 200 uM (each) deoxynucleoside triphosphate; 400 nM concentrations of primers CIF2F2, CIF2 R2 (for locus A), 200 nM concentrations of primers KDP F1, KDP R1 (for locus B) and 200 nM concentrations of primers RIF4 F3, RIF4 R9 (for locus E); 1.25 U of AmpliTaq. PCR amplifications were performed in a DNA Thermal Cycler 480 (Applied Biosystems) under the following conditions: initial denaturation at 94°C for 4 min followed by 30 cycles (denaturation at 94°C for 30 s, annealing at 53°C for 30 s, and extension at 72°C for 1 min) then final extension at 72°C for 4 min. The amplified PCR products were visualized by 2% agarose gel electrophoresis stained with ethidium bromide (0.5μg ml^−1^) and examined under UV trans-illumination (24).

### Statistical analysis.

Data entry and analysis were performed using SPSS version 16 (SPSS Inc., Chicago, IL, USA). The data were expressed as mean±SD and percentages. The sensitivity, specificity, positive predictive value (PPV), and negative predictive value (NPV) were calculated.

## RESULTS

The study included 234 subjects. They were 140 patients (59.8%) admitted mainly in surgical departments (42.9%). Health care workers were 94 (40.2%). *S. aureus* was isolated from 70 (29.9%) of the studied subjects (Table 2).

**Table 2.** Demographic and microbiological results of the studied subjects (n=234)

**Descriptive data**	**No.**	**(%)**
Gender		
Male	96	(41.02%)
Female	138	(58.9)
HCWs	94	(40.2)
Patients	140	(59.8)
Surgical wards	60	(42.9%)
Medical wards	40	(28.6%)
ED	40	(28.6%)
*Staphylococcus aureus* isolates	70	(29.9%)

ED: Emergency Departments

MRSA isolates were identified in the present study by cefoxitin screening test. The isolated MRSA represented 40% (28/70) from isolated *S. aureus.*

Isolated MRSA had high resistance rates for the used antibiotics. The most common resistance rates were for ciprofloxacin and chloramphenicol (57.1% for each), followed by resistance to erythromycin, clindamycin, trimethoprim-sulfamethoxazole and vancomycin (50% for each) (Table 3). MRSA were isolated mainly from 17.02% of the health care workers (16 isolates), while isolated from 8.6% of the patients (12 isolates).

**Table 3.** Antibiotics resistance among MRSA isolates (n=28).

**Antibiotics**	**No**	**(%)**
Gentamycin	12	(42.9%)
Ciprofloxacin	16	(57.1%)
Erythromycin	14	(50%)
Clindamycin	14	(50%)
Chloramphenicol	16	(57.1%)
Tetracycline	10	(35.7%)
Rifampicin	10	(35.7%)
Fusidic acid	12	(42.9%)
Imipenem	4	(14.3%)
Trimethoprim-Sulfamethoxazole	14	(50%)
Vancomycin	14	(50%)

**Fig. 1.**
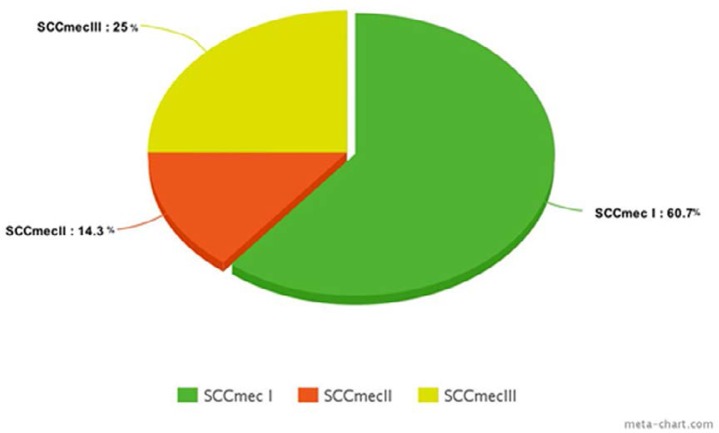
The frequency of SCC*mec* types among MRSA.

The frequency of SCC*mecA* of MRSA was 60.7% for type I, 25% for type III and 14.3% for type II (Fig. 1).

Chromogenic agar identified correctly MRSA isolates in 92.9% (26/28) and non MRSA isolates in 19.04% (8/42). PCR was positive in all isolates with resistance to cefoxitin disc (n=28) (Table 4).

**Table 4.** Comparison between MRSA and non MRSA isolates regarding the detection methods.

**Detection methods**	**MRSA (n=28)**	**Non MRSA (n=42)**
Chromogenic agar growth	26 (92.9%)	8 (19.04%)
PCR positive	28 (100%)	0 (0%)

The sensitivity, specificity, positive predictive value and negative predictive value of chromogenic medium compared to PCR in detection of MRSA were 92.9%, 84%, 76.5% and 95.5% respectively (Table 5).

**Table 5.** Statistical analysis of chromogenic medium compared to PCR for detection of MRSA

**Method of Detection**	**Sensitivity**	**Specificity**	**PPV**	**NPV**
ChromID medium	92.9%	84%	76.5%	95.5%

PPV: Positive predictive value

NPV: Negative predictive value

## DISCUSSION

Methicillin-resistant *S. aureus* (MRSA) presents a major problem in hospital infection and infection control because of resistance to multiple antibiotics. Furthermore, there are particular strains with an evident capacity for spread once introduced (2). The early identification of MRSA carriers is essential for the implementation of decontamination and isolation measures in order to prevent the dissemination of MRSA in health care facilities (16).

In the present study, the frequency of isolated MRSA among *S. aureus* as detected by cefexitin disc test was 40% (28/70). Variable prevalence of MRSA among *S. aureus* isolated from hospitals was reported and ranged from 5.5% up to 44.1% (25–27). The high prevalence of MRSA in the present study may be attributed to the inefficient knowledge and practice of infection control policies.

The most common resistance rates among MRSA isolates were for ciprofloxacin and chloramphenicol (57.1% for each), followed by resistance to erythromycin, clindamycin, Trimethoprim-sulfamethoxazole and vancomycin (50 % for each).

Udo et al. (2006) (28) reported similar findings with antibiotics resistance of MRSA to different types of antibiotics generations like chloramphenicol, trimethoprim, ciprofloxacin, aminoglycosides, erythromycin, tetracycline, fusidic acid, rifampicin, but they were susceptible to vancomycin. However, a recent study done by Hasan et al. (2016) (29) found 52% of MRSA isolates were resistant to vancomycin by the disc diffusion method. These results may be attributed to the presence of plasmid carrying multidrug resistance in MRSA to various types of antibiotics (30–32).

Emergence of vancomycin-resistant *S. aureus* (VRSA) may be due to the widespread use of vancomycin as being the first line drug in treatment of infections caused by MRSA, surgical procedures and involvement of health care workers infected or colonized with MRSA (29) which could explain the presence of high resistance rate to vancomycin in this study.

The main sources of MRSA causing hospital acquired infections are the colonized HCWs, patients and environmental objects (33).

In the present study MRSA was isolated significantly mainly from health care workers (17.02%). There are different reported rates for isolation of MRSA from health care workers that range from 0% to 59% (4, 34). This wide ranges of difference is attributed to many factors that influence the isolation rates of MRSA among these factors are the duration of the study, difference in the study design and the difference in isolation media used for each study, local infection control standards, and the local prevalence of MRSA (35–37).

From patients MRSA was isolated from 8.6%. Previous studies reported carrier rates of MRSA among patients to range from 10% up to 23.1% isolation rates (34, 36). The source of MRSA carrier in patients may be from community strains (36).

In the present study the most frequent SCC*mecA* genotypes were type I followed by type III and type II.

Previous studies reported different distribution of SCC*mecA* genotypes variance rates, in Brazil. Pacheco et al. (2011) (38) found that the main SCC *mec* types were mainly type IV (50%) and SCC*mec* type III (46%) and one had an indeterminate type among the hospital-acquired MRSA cases, while other study done by Caiaffa-Filho et al. (2013) (39) in the same country reported that SCC*mec* type II predominated followed by type IV and III. It is obvious that the genotypes of MRSA differ according to different hospitals rather than the difference in geographical locations.

PCR was positive in all isolates identified as MRSA by screening test. Previous study from Egypt identified positive PCR for *mecA* in (94%) of 29 isolates (40). The high frequency of positive PCR in the present study may be attributed to the use of multiple primers sequences to detect different SCC*mec* types.

The sensitivity, specificity, positive predictive value and negative predictive value of chromogenic medium compared to PCR in detection of MRSA were 92.9%, 84%, 76.5% and 95.5% respectively. It has been reported previously that though the molecular methods for detection of MRSA is superior to that of chromogenic media (41) regarding rapid results, this method has many limitations. The major disadvantage of PCR is its higher costs and its need for special expertise in its use. On the other hand the use of chromogenic media as screening method has good sensitivity and specificity beside its lower costs (42). The final decision which method to be used as a screening method depends upon several factors like hospital turnover, the cost of the culture and technical expertise. The low cost of chromogenic media encourage its use as a screening method for MRSA.

The subjects only represent the population in certain locality in Egypt. A larger study covering all Egyptian hospitals are mandatory to screen for the source of MRSA strains in hospitals especially among health care workers. Wide scale studies are recommended to detect the carrier rates of MRSA among both patients and health care workers in our locality. Thus we can decide the appropriate management of these conditions and whether or not to implant surveillance system to detect carrier rates among patients.

The present study highlights that MRSA carriage is common among health care workers in one Egyptian tertiary care hospital. Patients also have demonstrable carrier rates of MRSA. The major genotype of MRSA is belonging to SCC*mecA* genotype I followed by type III and type II. ChromID medium is an accurrate culture method for detection of MRSA compared to molecular method. Larger studies scales are required to demonstrate the impact of implementation of surveillance system for MRSA on infection control policies.
